# A Metapopulation Model of Tuberculosis Transmission with a Case Study from High to Low Burden Areas

**DOI:** 10.1371/journal.pone.0034411

**Published:** 2012-04-04

**Authors:** Roslyn I. Hickson, Geoffry N. Mercer, Kamalini M. Lokuge

**Affiliations:** 1 National Centre for Epidemiology and Population Health, Australian National University, Canberra, Australia; 2 School of Mathematical and Physical Sciences, University of Newcastle, Newcastle, Australia; McGill University, Canada

## Abstract

Tuberculosis (TB) is a growing problem worldwide, especially with the emergence and high prevalence of multidrug-resistant strains. We develop a metapopulation model for TB spread, which is particularly suited to investigating transmission between areas of high and low prevalence. A case study of cross-border transmission in the Torres Strait region of Australia and Papua New Guinea (PNG) is considered and a sensitivity analysis is conducted. We find that only 6 of the 50 parameters analysed are important to the cumulative number of clinically active TB patients in the entire region. Of these, only the detection rate in PNG is found to be an important intervention parameter. We therefore give insight into the extent the area with the high burden of TB (PNG in the case study) is dominating the TB dynamics of the entire region. Furthermore, the sensitivity analysis results give insight into the data that most important to collect and refine, which is found to be data relating to the PNG parameters.

## Introduction

Dual epidemics of HIV and tuberculosis (TB), and the associated multidrug-resistant TB (MDR-TB) are of increasing concern worldwide. TB is a disease of poverty [1] that mostly affects the developing world. However, there are many situations globally where a high burden community is in close proximity to a low burden one, for example, in Thailand-Burma [2], USA-Mexico [3], and in the Papua New Guinea (PNG)-Australian border regions. In Fact, approximately 25% of Australia's MDR-TB cases occur in the Torres Strait Islands (TSIs) region between mainland Australia and PNG [4]. The aim of the case study is to identify the factors that most affect TB transmission in the Torres Strait region.

The aetiology of TB is complicated with different rates of progression to, and different states of, the clinically apparent disease. Approximately 5–10% of those infected with TB progress to clinically apparent TB within 2 years [5]. The remainder stay latently infected, with 5–10% progressing to clinically apparent TB over a 20 year period [6]. Those with clinically apparent TB are further divided into extra-pulmonary and pulmonary TB, with the pulmonary form being the main source of TB transmission. Therefore, for the purposes of our work, individuals with pulmonary TB are ‘infectious’ and individuals with extra-pulmonary TB are ‘non-infectious’. Note those in the ‘pulmonary’ category may also have extra-pulmonary TB, but the reverse is not true.

The control of TB spread is of paramount importance, and the main intervention strategy adopted by the World Health Organisation Stop TB program is ‘Directly Observed Treatment Short course’ (DOTS) [1] provided through National Tuberculosis Programs. These provide the bulk of TB detection and treatment services in high burden countries. Therefore, when considering interventions, we concentrate on the DOTS program, and do not explicitly consider other interventions here. Further information is provided about the DOTS intervention program in Section 2.1.1.

A significant aspect of this work is the use of a metapopulation model to capture the disease dynamics across regions with different transmission rates or burdens of TB. The metapopulation model overcomes the assumption of homogeneous mixing between these different regions, which is implicit in compartment models with a single population. This paper builds on the model in Hickson *et al.* [7], which considers treatment of TB in PNG. Although metapopulation models of TB have previously been considered [8], [9], the models have been relatively simplistic, not taking into account both pulmonary extra-pulmonary TB, and have not explicitly taken into account the DOTS program which is considered here. Furthermore, although the model presented here can be used for two regions of any burden, which are interested in transmission from regions of high to low burden. In particular, we focus on transmission from PNG to the Australian TSIs, which has a large health care gradient. Therefore, we build a model appropriate to our scenario of interest and do not use and already established, but not entirely relevant, model. The model presented here will form the baseline to compare extensions including MDR-TB and HIV to determine their related transmission dynamics and impact.

Although the TB transmission dynamics are thought to be driven by the high burden community, it is not known to what extent, or what disease and intervention aspects the dynamics are most sensitive to. In Section 2.1 we construct a compartmental metapopulation model to increase understanding of the transmission dynamics. The model is then applied to the Torres Strait region of Australia and PNG, and a sensitivity analysis of the model parameters is conducted in Section 2.2.

## Methods

### 2.1 The model

We construct a relatively simple model for drug sensitive TB that ignores MDR-TB and HIV. Although these are important factors for TB transmission, an understanding of the dynamics of TB is possible and more straightforward with a simpler model. Future work will extend this model to include MDR-TB and then HIV to determine their related transmission dynamics and impact by comparing the results to the baseline presented in this article.

The model is a population level compartment model coupled with a metapopulation model. This is essential as we are interested in the transmission dynamics of discrete populations that have very different healthcare systems and TB prevalences (for example, PNG nationals and Australian Torres Strait Islanders). In a compartment model the population is divided into discrete compartments each representing a different stage of the disease, or treatment. The metapopulation aspect divides the population into a number of regions, 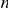, and the number of sub-populations is then 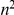 to allow for travel between each region. The (local) disease model is hence applied in each of the sub-populations, and described in Section 2.1.1. The extension to metapopulation is then described in Section 2.1.2.

#### 2.1.1 (Local) disease model

**Figure 1:**
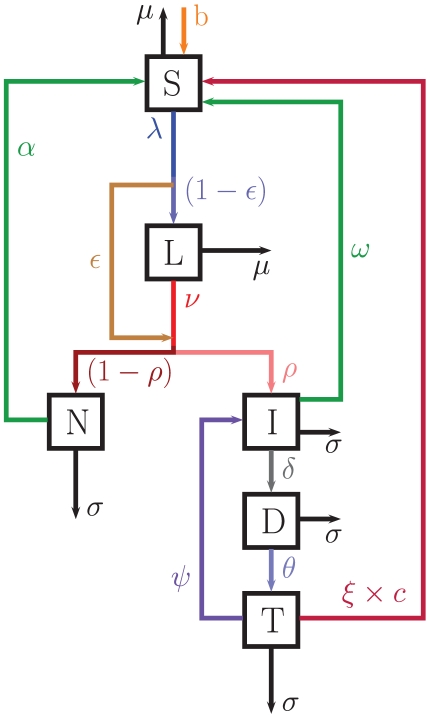
The (local) disease model. S = Susceptible, L = Latently infected, N = non-infectious clinically apparent TB, I = infectious clinically apparent TB, D = Detected but not yet treated, T = undergoing Treatment but still infectious. The parameters are described in the text and summarised in the table in File S1. Here 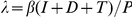.

Here we are effectively looking at a single sub-population group, which will be expanded to the metapopulation in Section 2.1.2. TB is a many-faceted disease, and hence the (local) disease model reflects this. Figure 1 depicts the compartment model for drug sensitive TB, based on the model analysed in Hickson *et al.* [7]. Figure 1 is hence similar to Figure 1 in Hickson *et al.* [7]. The arrows show possible progression paths through different stages of the disease. Here we use the term “clinically apparent” to refer to patients where the level of TB is able to be detected, but may or may not be pulmonary and hence easily infectious to other patients.

The model is composed of 6 compartments, 4 for the disease itself (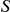, 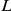, 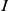, 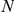), and 2 for the interventions (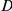, 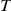). The 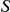 compartment represents those susceptible to TB, 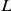 is those who are latently infected with TB, 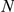 is those clinically apparent with extra-pulmonary infection, and 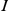 is those clinically apparent with a pulmonary infection, and so are able to infect others. An individual can become clinically apparent by essentially skipping the latent class with probability 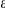, which is ‘fast’ TB, or first become latently infected and have ‘slow’ TB with probability 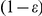. It is assumed that once an individual is latently infected, they remain so until either death or they become clinically apparent. Once clinically apparent, it is possible for someone to spontaneously clear TB from their system, with rate 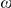. Those with clinically apparent TB have a higher death rate, 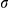, than the natural death rate of the susceptible and latently infected, 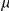.

Infection and clinically apparent disease does not confer immunity to future disease in the case of tuberculosis. Difficulties in differentiating TB reinfection with TB reactivation means quantification of the exact proportion of disease attributable to each category in those who have previously had active tuberculosis is not possible, and unnecessary in developing a model of transmission. However, it is reasonable to assume that those who have previously had active disease have at least a similar risk of developing active tuberculosis to those who have never previously had active disease [10], [11].

The remaining population compartments are for the DOTS intervention program, which only those with pulmonary TB (I) progress to. The WHO definitions for treatment outcomes are used. The first intervention compartment is for individuals who have been detected (D), and the second is those who are undergoing treatment, but are still infectious (T). It is assumed that everyone detected is (eventually) treated. A default rate from treatment (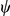) has been included, and it is assumed that once someone defaults they return to the ‘I’ class. The WHO definition of ‘defaulted’ is “a patient who has interrupted treatment for two consecutive months or more” [12]. Furthermore, those undergoing treatment are cured with the rate 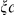, where 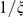 is the duration an individual is infectious whilst undergoing treatment, and 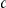 is the proportion of treated cases successfully cured. The WHO definition of ‘cured’ is a “former smear-positive patient who was smear-negative in the last month of treatment, and on at least one previous occasion” [12]. There is an implicit return of those who have failed treatment back into the treatment compartment (T), with rate 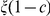. The cure rate from the N class is 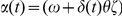, where 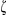 is the completion rate of treatment for extra-pulmonary TB cases. This comes under the WHO definition of ‘completed treatment’ which is “A patient who has completed treatment but who does not meet the criteria to be classified either as a cure or a failure” [12]. Therefore, the DOTS program for extra-pulmonary TB is included in the recovery from the ‘N’ compartment. A natural cure rate was not included for either the detection or treatment compartments (D & T) as comparatively short average durations are expected for these.

The governing system of ordinary differential equations is
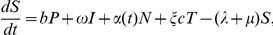


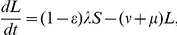


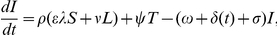


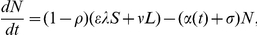


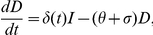


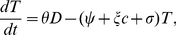
(1)where 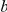 is the birth rate of susceptibles into the population, 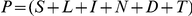 is the total population, the force of infection 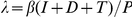, and 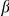 is the transmission rate. A full list of parameters and their definitions is given in the table in File S1.

#### 2.1.2 Metapopulation extension

The (local) disease model presented in Section 2.1.1 assumes homogeneous mixing within the entire population of interest. We are interested in situations where there are dramatic differences in burden of TB across a region, which is an outcome of a metapopulation structure. That is, there is reduced mixing of individuals, for example across a border, and hence homogeneous mixing is not an appropriate assumption. The differences in burden across a region are usually a result of different transmission structures and health care access. Therefore, we construct a metapopulation model where the (local) disease model is applicable for each sub-population and cross-border movement is explicitly modelled.

Metapopulation models such as this are well established, see for example Keeling and Rohani [13]. The metapopulation model has the same compartment structure as the single population model, with Susceptibles (S), Latently infected (L), clinically apparent extra-pulmonary TB (N), clinically apparent pulmonary TB and so able to Infect others (I), infectious and have been Detected (D), and currently undergoing treatment (T).

The metapopulation model is split into two distinct categories, those of a population group in their home region (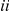), and those visiting region 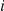 from population 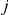. Homogeneous mixing occurs between the sub-populations in a region. The rate of change of population groups in their home region are given by
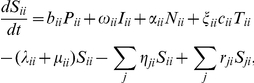


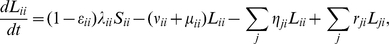


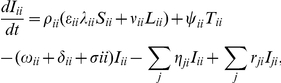


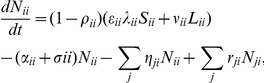


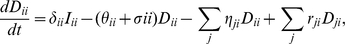


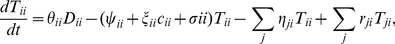
(2)where the force of infection 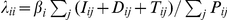, 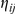 is the rate of departure from region 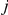 to region 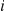, and 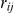 is the rate of return from visited region 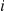 to home region 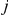. For example, the term 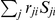 is the sum of those who return to their home sub-population (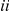) from all other sub-populations. Similarly, the rate of change of the population groups not in their home region are governed by
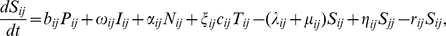


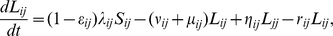


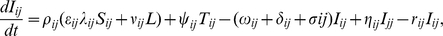


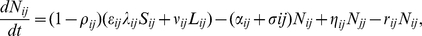


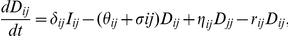


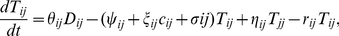
(3)where 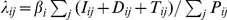. This force of infection hence assumes the transmission rate is driven by the region, not the sub-population, though this is further discussed in Section 2.2.2. Note the difference in sign for the ‘departure’ and ‘return’ terms. Departure is defined as departure from the individuals ‘home’ region, and hence adds to other regions, and vice versa. Equations (3) are simpler than Equations (2) due to the assumption that each individual returns to their home region before departing for another region.

**Figure 2:**
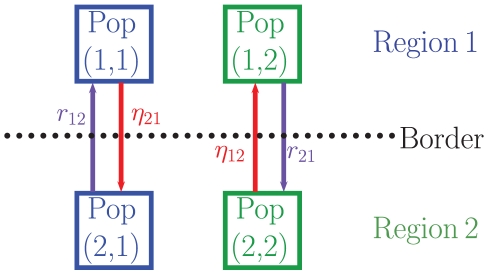
Schematic of sub-populations in the metapopulation, for a 2 region example. Note homogeneous mixing occurs within each side of the border.

For illustrative purposes, a two region metapopulation schematic is depicted in Figure 2. Homogeneous mixing occurs each side of the border, for example between sub-populations (1,1) and (1,2), where the first coordinate denotes the current location, and the second which region the population is originally from. For an 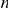 region metapopulation model there are 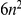 different compartments to consider, since taking into account individuals visiting other sub-populations there are 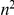 population groups and 6 compartments within each of these to discriminate the disease status. Although a 2 region model has been depicted, Equations (2) and (3) are for a general 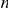 region model.

In Figure 2, 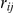 denotes the rate of return from the visited region, 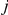, to the home region, 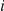, and 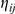 the rate of departure from the home region, 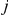, to the visited region, 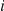. The average duration of stay, 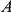, is given by [13] (pg. 245)
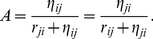
(4)We have assumed movement between regions in the metapopulation model is for short-term visits only, and hence there is no permanent migration. Keeling and Rohani [13] suggest this is a reasonable assumption, as permanent migration is not an epidemiologically significant force (pg. 242). This is particularly true in the case study considered below as permanent migration is minimal in that region.

### 2.2 A case study: Papua New Guinea & the Torres Strait Islands

Papua New Guinea (PNG) is considered a high burden TB country, with an estimated average prevalence of 430/100,000 population, and incidence of 250/100,000 population in 2007 [12], [14]. There is also believed to be a high prevalence of multidrug-resistant TB [12] (pg. 36). The Torres Strait Islands (TSIs) are part of Australia, which is a low burden TB country with an average prevalence and incidence of 6/100,000 population in 2007 [12] (Table 1, page vii).

**Table 1:** The relative sensitivities of the intervention parameters at ‘2032’.

Sensitivity order	Total population^1^		Australian population^2^	
1.	(1,1)^3^ 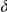	(−0.155)^4^	(2,2) 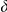	(−0.122)
2.	(2,1) 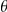	(−0.038)	(2,2) 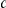	(−0.105)
3.	(1,1) 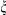	(−0.037)	(2,2) 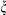	(−0.092)
4.	(1,2) 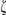	(0.035)	(1,1) 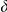	(−0.039)
5.	(1,2) 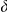	(0.033)	(2,2) 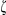	(−0.031)
6.	(2,1) 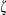	(−0.028)	(1,1) 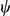	(0.025)
7.	(2,2) 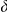	(−0.026)	(1,2) 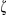	(0.022)
8.	(1,2) 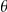	(−0.023)	(2,1) 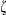	(−0.021)
9.	(1,1) 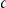	(−0.022)	(1,2) 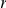	(−0.020)
10.	(1,1) 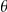	(−0.021)	(2,1) 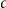	(−0.018)

At the closest point, the TSIs are less than 5 kms from the Western Province of the PNG landmass. Furthermore, there is an international treaty which allows free travel between certain areas of the TSIs and PNG for ‘traditional activities’, such as trade and cultural activities. The border is therefore quite porous, with over 59,000 cross-border movements recorded in 2008–09 for traditional activities, and 98% of these movements were made by PNG nationals [15]. The average length of stay is thought to be about a day [15], although actual data has not yet been published.

The Western Province of Papua New Guinea mostly consists of small, isolated communities, with the average population density of 1.6 people per square kilometre [16]. Only 17.2% of the population in the Western Province lives within 5 kms of a national road [16]. The South Fly region is the southern most region of the Western Province, there are only 2 medical officers for this district, and most transport is via waterways [16]. Therefore, we assume interaction between the South Fly communities in the Treaty Zone and other PNG communities is limited to the point of virtual non-existence. The bulk of the TB services in the Western Province are in the capital, Daru, which is some distance from South Fly. Therefore, TB services in Australia are more accessible to those in the South Fly in terms of cost and distance to travel than TB equivalent services in PNG. As a result, PNG nationals have historically had access to health care in the Australian TSIs. For the purposes of the metapopulation model, the South Fly district of PNG is region 1 and the Australian TSIs are region 2. Thus, sub-population (1,1) refers the PNG nationals in PNG, and (2,2) to Australians in the TSIs.

Approximately 25% of MDR-TB cases in Australia occur in the TSI region [4]. A single case of MDR-TB costs approximately USD$250,000 to treat in the US [17], and similar costs are expected in Australia although there is limited data on this. Hence there is a large economic impact associated with not only the rising TB incidence, but the high proportion of MDR-TB. Indeed, due to this high cost, the Australian government is currently in the process of ceasing treatment of PNG nationals for TB in Australia [18].

The DOTS intervention program in PNG started in 1997 [19], hence the detection rate, 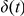, for sub-population (1,1) is taken to be non-zero from 1997. Similarly, the DOTS intervention program started in Australia in 1990, hence for sub-populations (2,2), (2,1) and (1,2) 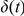 becomes non-zero from 1990. Furthermore, as initial increases in detection rates are relatively easy and further increases become more difficult, 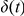 increases using a logarithmic function for all sub-populations, as detailed in the Equation in File S1.

The PNG-TSI area is potentially a major source of drug sensitive and MDR-TB into Australia, and is therefore of interest to health departments in Australia. Our focus is hence on the cross-border transmission dynamics of TB from PNG to the Torres Strait Islands. This issue is highly charged politically, and the WHO is currently negotiating a workable compromise between the Australian and PNG health systems [18], [20]. The aim of this case study is to identify the factors that most affect cross-border TB transmission in this region.

We perform a sensitivity analysis to determine the relative effect of the parameters in the model. This allows us to identify which parameters data refinement should concentrate on determining as accurately as possible, which is particularly important given the limited data, as discussed in Section 2.2.3. The relative effect of parameters also gives insight into how to improve intervention programs most effectively, and which assumptions are most affecting the model outcomes. The parameter values, units, assumptions, and references for this 2-region case study are presented in the table in File S1.

#### 2.2.1 Metapopulation regions and sub-populations

Although previously mentioned, the metapopulation regions and sub-populations are summarised here to aid readability. The South Fly district of PNG is defined as region 1 and the Australian TSIs as region 2 in this case study. Hence PNG nationals in PNG are sub-population (1,1), and Australians in the TSIs are sub-population (2,2). Furthermore, PNG nationals in the Australian TSIs are sub-population (2,1), and Australians in PNG are sub-population (1,2).

#### 2.2.2 Parameter assumptions

We do not have data for the sub-populations travelling in different regions. That is, for sub-populations (1,2) and (2,1). Therefore, assumptions were made to determine the parameter values in terms of sub-populations (1,1) and (2,2). These assumptions and hence the nature of the data that would be required are outlined in this section.

We assumed that Australians travelling to PNG, sub-population (1,2), have the same parameter values as Australians in the TSIs. This effectively means the travel times to PNG are short, and their access to health care is not altered. However, we have assumed the transmission rate is dependent on location and not nationality, and therefore 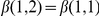 and similarly 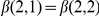. The effect of this assumption on the sensitivity analysis was explored, and no qualitative difference was found when 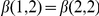 and 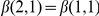. Furthermore, having transmission rates vary from those in compartments ‘I’, ‘D’ and ‘T’ was explored. However, a sensitivity analysis showed the transmission rates specific to population compartments ‘D’ and ‘T’ were unimportant, and so a single transmission rate is used.

The assumptions surrounding PNG travellers to Australia, sub-population (2,1), are more complicated. First we assume the disease progresses in travellers from PNG at the same rate as locals in PNG. That is 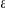, 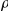, 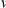, and 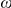 are equal for sub-populations (1,1) and (2,1). Second, we assume the component of the death rate 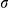 due to TB in the TSIs is half of that in PNG. This is partly due to the assumption of 1 day average duration in the TSIs, and partly due to better access to health care should death be imminent in the TSIs. We assume that PNG nationals in the TSIs are detected, but not as effectively as locals. The difference is not known, therefore we assume detection is half as effective, so 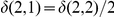. Similarly, we assume the delay between detection and treatment is twice as long for travellers in the TSIs than for locals. We assume the default rate from treatment is halved for sub-population (2,1) than for (1,1). That is, 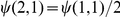. This is partly because if travellers are seeking health care in the TSIs they are less likely to default, and partly because the health care centre in the TSIs is closer than the one in the PNG town of Daru. However we assume half as many PNG nationals are likely to complete treatment if they have extra-pulmonary TB than TSI locals, so 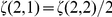. Finally, since we are assuming treatment is occurring under the TSI health care system, the duration an individual is infectious whilst undergoing treatment and the proportion of treated cases successfully cured (if treatment is finished) are the same as for locals. That is 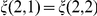 and 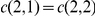.

#### 2.2.3 Model calibration

To determine the transmission rates, various model outputs were compared to data from the World Health Organisation (WHO) [12]. The WHO collects and estimates data on the incidence, prevalence, and mortality rate of TB, and therefore all of these were used to calibrate the model. The calibration was performed against the PNG and Torres Strait Island regions of the model, as they have significantly different burdens of TB.

There are several issues with the data from the WHO with regards to PNG. First, the data for PNG is reported only at the national level, whereas South Fly is better connected to the Australian Torres Strait Islands than the rest of PNG. In addition, the South Fly demographic is more isolated, and is believed to have a higher average burden than the national average. The isolation also inhibits data collection, so the average burden is likely to be significantly underestimated. Furthermore, since insufficient data were available for PNG, the incidence was assumed to follow a flat trend going through the best estimate [21]. Therefore, the model probably underestimates the transmission rates within PNG, and therefore underestimates the effect of control strategies.

The full metapopulation model, Equations (2) and (3), were solved using ‘ode15s’ in MATLAB [22]. The model was run with a single infectious case in PNG from ‘1800’, until the epidemic stabilised, and the transmission rates were determined so that the incidence, prevalence, and death rate matched data available from the WHO [12] for PNG and Australia, for the appropriate sub-populations. Furthermore, the total population in 2009 for PNG and 2006 in Australia were matched. The initial conditions for the intervention analysis were subsequently determined by the quasi steady state of the ‘1800’ run. For all results the model is run from 1950, as this allows the model to settle before the intervention begins in 1990, and covers the period of interest (1990–2032).

#### 2.2.4 Sensitivity analysis

Conducting a sensitivity analysis is important in terms of both data collection and refinement, and in the context of understanding and informing the control of TB, by determining which parameters are most influential on the spread. This is especially true in this case, given the limited and problematic data available and the large number of assumptions subsequently made. The sensitivity analysis requires the model to be run many times with different parameter values drawn from appropriate distributions. Latin Hypercube Sampling (LHS) is used for efficient sampling of the parameter values, in conjunction with Partial Rank Correlation Coefficient (PRCC) multivariate analysis to determine which parameters most influence the disease progression, as previously conducted [23]–[26]. Three thousand samples are used for the LHS.

The combination of LHS and PRCC is a standard technique that has been used widely for sensitivity analyses of models [23]–[26]. A positive coefficient indicates a positive correlation between the input parameter and the output of interest, whilst a negative coefficient indicates a negative correlation. A rank with a magnitude close to 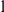 indicates a dominant parameter, while the least important parameter rank coefficients are near zero. The PRCC analysis requires a monotonically increasing function, hence the cumulative number with clinically apparent TB (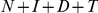) is used.

Several parameters are excluded from the sensitivity analysis as they are not of interest for our study: the birth rates, 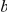; the natural death rates, 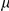, and the total population, 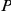. The parameters for sub-populations (2,1) and (1,2) are varied independently of the parameters for sub-populations (1,1) (‘PNG’) and (2,2) (‘TSI’), and hence there are a total of 50 parameters of interest. In keeping with previous work [7], [26], triangular distributions are assumed for all parameters, with a range of 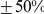 the value presented in the table in File S1. To explore the stability of the PRCC results to the width of the parameter distribution, ranges from 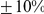 to 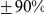 were also explored. The results were qualitatively similar, with relatively small quantitative differences in the rank.

## Results

**Figure 3:**
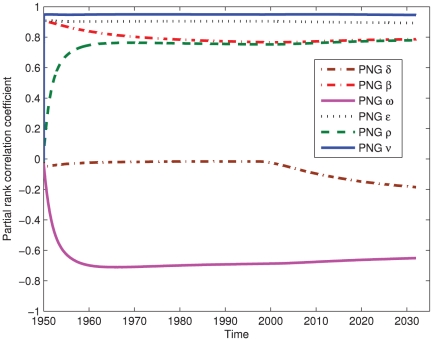
Sensitivity analysis of the model parameters, comparing the relative monotonicity of the cumulative number with active TB (I+N+D+T) for all sub-populations with various parameters. ‘PNG’ refers to sub-population (1,1) parameters.

Three separate analyses were performed. First, the outcome on the total of all sub-populations is depicted in Figure 3. Second, the outcome on the total PNG sub-populations, (1,1) plus (2,1), was determined. There was little qualitative and small quantitative differences in the outcome of the sensitivity analyses of the total or PNG-only populations, which is discussed later. This is due to the dominance of the PNG dynamics on the overall incidence of TB in the entire region. Finally, the outcome on the total Australian sub-populations, (2,2) plus (1,2), was determined. There are 11 parameters that the cumulative number of TB cases in Australia were sensitive to, with 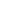
PRCC values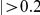. Hence the analysis for Australian sub-populations is confined to Table 1, where the sensitivity of intervention parameters is considered.

Figure 3 depicts the sensitivity analysis for the total population, where parameters with low sensitivity, 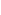
PRCC values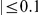 at the final time, have been excluded from the figure for clarity. As mentioned in Section 2.2.3, the model is run from 1950 to allow it to settle before the introduction of the DOTS intervention programs. Figure 3 illustrates that the sensitivity analysis has reached a quasi steady state before 1990, when the DOTS program began in the TSI region.

Interestingly, only 6 of the 50 parameters analysed have a sensitivity measure 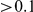, as depicted in Figure 3. Note all 6 are ‘PNG’ values, and the smallest PRCC value of the disease parameters is about 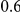. The PNG detection rate, 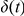, is taken as non-zero at 1997, and is slowly increasing in importance with time. This result suggests an increase in DOTS coverage in PNG by even a small margin will result in a significant decrease in incidence and prevalence of TB in both PNG and the Australian TSIs.

The most sensitive intervention parameters for the total and Australian-only populations are shown in Table 1. The total population refers to all four sub-populations, whereas the Australian-only population is the sum of (2,2) and (1,2). The travel rates between the populations are also included as they may be influenced (for example by border closure or changes to the treaty conditions). Of particular note is the importance of the detection rate in PNG (‘(1,1) 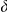
’) for the total population, with a PRCC of approximately 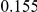. The completion rate from treatment, 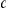, is also important, in PNG for the total population and in Australia for the Australian population.

## Discussion

Figure 3 shows the cumulative number of TB cases is more sensitive to the disease parameters (in particular 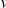, 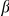, 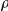, 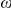, and 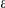) than the ‘intervention’ parameters (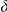, 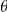, 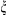, 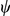, 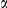, 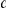, 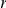 and 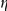). However, it is far easier to influence the intervention parameters than the disease parameters. Therefore, data collection and refinement should concentrate in PNG.

PNG presents challenges for all health programs, including tuberculosis control, that include very low population density, difficult access due to rugged terrain, limited resources, and in particular human resource shortages [27]. Due to these issues, PNG continues to perform relatively poorly in tuberculosis control. The sensitivity analysis results outlined in Table 1 show that the detection rate in PNG, 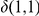, is the most important parameter in terms of the cumulative number with clinically apparent TB for the entire region. However, access to treatment is limited on the PNG side of the border, with the closest hospital in Daru, which is several days travel from the South Fly district. Clinical access in the Torres Strait Islands for PNG nationals is therefore important for the control of TB in the entire region.

The assumption was made that the transmission rate is dependent on current location and not the region of origin. This assumption was switched, such that the transmission rate is dependent on the region of origin and not the current location. There was no qualitative and no significant quantitative effect on the sensitivity analysis with this alternative assumption.

Estimated levels of BCG coverage in PNG are relatively high [28]. However, the main impact of BCG is on mortality due to childhood TB, and in a more limited way, extra pulmonary TB in immunocompetent individuals. Its impact on tuberculosis transmission in the population as a whole is probably minimal [29]. Therefore, parameter estimates used in the models, which are derived from contexts with similar levels of BCG coverage, were assumed to account for the impact, if any, of BCG vaccination.

Previous models of TB transmission dynamics have been used to explore implications of endogenous reactivation of latent infections for public health interventions [30], have considered drug-resistant TB [31], [32], and others have considered co-epidemics of HIV and TB [33], [34]. Some models [35], [36] consider both co-epidemics of HIV and TB, and drug-resistance through a failed treatment group, but do not explicitly consider a drug-resistant TB strain. Basu *et al.* [37] also consider both co-epidemics of HIV and TB and drug-resistance, but their model focuses on an HIV intervention in Botswana.

The model presented here only explicitly considers drug sensitive TB. Building multidrug-resistance and HIV into the model is the subject of future research. However, their inclusion is unlikely to change the sensitivity analysis demonstrating the disease dynamics are being driven by the high burden region, which is PNG in our case study. Thomas *et al.* [26], consider the spread of TB in the Western Province of PNG. Although they included both HIV and MDR-TB in their ‘detailed’ model, they did not include compartments to analyse treatment, and consider only the PNG region. Thomas *et al.* [26] showed the results of their detailed model are qualitatively similar to the results where only drug sensitive TB is considered. However, a notable difference was a lower predicted prevalence for the detailed model after the introduction of DOTS in 1997. This could be due death rates of those with HIV and MDR-TB being higher than the drug sensitive death rate, leading to a general reduction in TB incidence. Therefore the effect of including MDR-TB and HIV on the results will not be straightforward.

The model presented here is applicable in other areas of high to low burden transmission, particularly across national borders from developed to developing world countries. For example, a similar dynamic occurs in the Thailand-Burma region [2] and in the USA-Mexico region [3]. Furthermore, although only 2 regions are considered here, the model outlined in Section 2.1 is applicable to any number of areas. The model presented here can be used for two areas of any burden, as long as the parameters reflect the properties of each region. That is, although we have used an example from a high burden to a low burden area, the model is equally valid for two high burden areas or two low burden areas.

The case study considers a scenario where the average length of stay is 1 day, based on advice from the Department of Immigration and Citizenship [15]. If the average length of stay is increased from 1 day, the prevalence and incidence of TB will increase in the community with the lowest burden. The sensitivity analysis identifies factors which most contribute to the cumulative number of people with clinically apparent TB, pulmonary and extrapulmonary. If the average length of stay is increased to 2 weeks, there is not qualitative difference in the outcome. However, if PNG nationals stay for greater than 1 day on average, the overall effect will be a significant increase in incidence and prevalence in the Australian TSIs.

### 3.3 Conclusions

This analysis shows that TB in Papua New Guinea nationals is driving the TB dynamics in the entire Torres Strait region. Although this result was expected, the extent of the dominance is now better understood. The most important disease parameters are those within PNG, and the most important intervention parameter is the detection rate in PNG. However, given the challenges PNG faces for health programs, and that TB services in Australia are more accessible to those in the South Fly district, treatment and interventions performed on PNG nationals while visiting the TSIs is very important for the control of TB in the entire region. For interventions in PNG to have greater impact on disease burden their effectiveness must be increased. That is, less delay between detection and treatment, a lower default rate from treatment, and a higher completion rate. The estimated transmission rate in PNG is also significantly higher than that of Australia, hence interventions and health care conditions need to be improved in PNG to reduce incidence and eventually prevalence of TB in the Torres Strait region.

The effect of varying the current intervention program involves subtleties in parameter variations, as suggested by the discussion of model assumptions in Section 2.2.2. Future work will explore the effect of intervention programs on incidence and prevalence of TB in the entire Torres Strait region. A cost-benefit analysis will then be conducted to determine the most effective intervention scenarios. Future work will then extend the current model to include MDR-TB and then HIV to determine their effect on the TB dynamics. Other interesting factors to explore include the effect of smoking and diabetes on TB, although these are less prominent in PNG than MDR-TB and HIV.

## Supporting Information

File S1 **A full list of the model parameters and their definitions is given in the table in File S1, the time dependent function for the detection rate is given in an equation, and the appropriate references for the parameter values are given.**
(PDF)
