# Supplementary material for: A Metapopulation Model of Tuberculosis Transmission with a Case Study from High to Low Burden Areas
Source: PLoS One. 2012 Apr 4;7(4):e34411. doi: 10.1371/journal.pone.0034411 (PMC3319591; doi:10.1371/journal.pone.0034411)
Supplement: File S1 — A full list of the model parameters and their definitions is given in the table in File S1, the time dependent function for the detection rate is given in an equation, and the appropriate references for the parameter values are given. (PDF) [file pone.0034411.s001.pdf]

File S1: Parameter descriptions

Parameter	Description	Value (1,1)	(2,2)	(2,1)	(1,2)	Units	References	Notes and assumptions
b	Natural birth rate	0.0317	0.0317	(1,1)	(2,2)	/year	[1]	
μ	Natural death rate	0.0159	0.0143	(1,1)	(2,2)	/year	[2], [3]	Inverse of lifespan, averaged over female and male. Assuming exponentially increasing populations.
σ	TB positive death rate	0.0165	0.01431	(1,1)/2	(2,2)	/year	[4] Table 1, pg. xii	Calculated as $\mu +$ TB induced death rate.
ϵ	Proportion of people progressing to active TB within one year of infection	0.0375	0.025	(1,1)	(2,2)	None	[5]	Estimate, noting 5–10% infected with TB are expected to progress to active TB within 2 years.
ρ	Proportion developing infectious TB	0.7	0.7	(1,1)	(2,2)	None	[6]	Assuming TSIs=PNG.
ν	Rate of progression to active TB	0.0018	0.0013	(1,1)	(2,2)	/year	[7]	2.6% progression in 20 years.
ω	Natural cure rate of active TB	0.3168	0.3190	(1,1)	(2,2)	/year	[8]	Assumed equal for the 'N' and 'I' compartments.
β	TB transmission coefficient	30	6	(2,2)	(1,1)	/year	N/A	Estimates. Assuming the transmission coefficient is locally driven and equal transmission from I, D, & T.
$\delta(t)$	Case detection rate under DOTS (pulmonary cases)	See Equation (5)				/year	N/A	Time (t) dependent function starting at zero[1] and logarithmically increases to $\delta_{\max}$.

[1] Applies after the introduction of DOTS to PNG in 1997 (page 45 of [9]) and in the Torres Strait in 1990.

$\delta_{\max}$	Maximum case detection rate under DOTS (pulmonary cases)[2]	0.15	0.49	(2,2)/2	(2,2)	/year	[4] Table 33, page 69	Assuming not detecting as many visitors as locals in TSIs. Overestimated incidence for Australia means this is an underestimate of detection.
$1/\theta$	Delay between detection and treatment	4	1	(2,2)*2	(2,2)	Weeks	[10]	Assuming larger delay between detection and treatment in PNG. Conservative as Western Province of PNG isolated.
$1/\xi$	Duration person infectious whilst undergoing treatment	5.5	5.5	(2,2)	(2,2)	Weeks	[11]	NOT the 6 months required for treatment as not infectious for that long.
c	Proportion of treated infectious cases successfully cured	0.59	0.13	(2,2)	(2,2)	None	[4] Table 35, pg. 72	
ψ	Default rate from treatment	0.21	0.01	(1,1)/2	(2,2)	/year	[4] Table 35, pg. 72	Assuming PNG nationals less likely to default if treated in TSI.
ζ	Completion rate of treatment from 'N'	0.15	0.72	(2,2)/2	(2,2)	/year	[4] Table 35, pg. 72	Assuming those visiting TSIs for treatment from PNG are half as likely to complete treatment.
$\alpha(t)$	Combined treatment and natural cure rate for non-infectious cases	Calculated as $\omega + \delta(t) \times \xi \times \zeta$				/year	N/A	Time (t) dependent, since it's a function of $\delta(t)$.
η	Rate of departure from 2nd index to 1st	0	0	0.57	0.07	/year	[12]	Calculated using number of departures divided by population of each region.

[2] Annual new smear-positive notifications under DOTS divided by estimated annual new smear-positive incidence, 2007.

r	Rate of return from 1st index to 2nd	0	0	188.33	198.24	/year	[12]	Calculated using $r = \eta \times [P/(M \times A) - 1]$, where P is the total population (in 2008/09), M is the number of movements (people/year), and A is the average duration of stay, assumed here to be 1 day.
P	Total population	46, 537[d]	8,576[e]	N/A	N/A	People	[13] & [14] (pg. 2)	[d]South Fly District of PNG only in 2009. [e]Estimated population in 2006.

Table 2. Table of parameter values and assumptions. (1,1) refers to Papua New Guinea (PNG) citizens in PNG; (2,2) to Australians in the Torres Strait Islands (TSIs); (2,1) to PNG nationals in TSIs; and (1,2) to Australians in PNG.

The detection rate, $\delta(t)$, is a time dependent function, staying at zero until the DOTS program begins and increases to the current value,

$$\delta(t)(i,j) = \begin{cases} 0 & t < t_\delta(i,j)\,, \\ \dfrac{\delta\mathrm{max}(i,j)}{\ln(2007 - t_\delta(i,j))} \ln(t - t_\delta(i,j)) & t_\delta(i,j) \le t < 2007\,, \\ \delta\mathrm{max}(i,j) & t \ge 2007\,, \end{cases} \tag{5}$$

where $t_\delta(1,1)$ =1997 in PNG, $t_\delta(2,2)$ =1990 for the Torres Strait Islands, and the travellers are assumed to have the earliest start time, so $t_\delta(1,2)$ =1990= $t_\delta(2,1)$.

References

1. United Nations Population Division (2008). World population prospects: The 2008 revision population database. http://esa.un.org/unpp/.

2. Lahmeyer J (2004). Population statistics. http://www. populstat.info/ (retrieved 11 Feb 2011).

3. Australian Bureau of Statistics (2010). The health and welfare of Australia's Aboriginal and Torres Strait Islander Peoples. http://www.abs.gov.au/AUSSTATS/abs@.nsf/lookup/4704.0Chapter218Oct+2010, (accessed 10 March 2011).

4. World Health Organisation (2009) Tuberculosis control in the Western Pacific region. Technical report, World Health Organization.

5. Porco TC, Small PM, Blower SM (2001) Amplification dynamics: Predicting the effect of HIV on tuberculosis outbreaks. J Acq Imm Def Synd 28: 437-444.

6. Yang Z, Kong Y, Wilson F, Foxman B, Fowler AH, et al. (2004) Identification of risk factors for extrapulmonary tuberculosis. Clin Infect Dis 38: 199-205.

7. Blower SM, McLean AR, Porco TC, Small PM, Hopewell PC, et al. (1995) The intrinsic transmission dynamics of tuberculosis epidemics. Nature Medicine 1: 815-821.

8. Tiemersma EW, van der Werf MJ, Borgdorff MW, Williams BG, Nagelkerke NJD (2011) Natural history of tuberculosis: Duration and fatality of untreated pulmonary tuberculosis in HIV negative patients: A systematic review. PLoS ONE 6: e17601.

9. World Health Organisation (2003) Tuberculosis control in the WHO Western Pacific region. Technical report, World Health Organization. http://www.wpro.who.int/NR/rdonlyres/F9E1ED36-B3E6-4E5C-A6F3-7FD65DB760C8/0/tbcontrol2003.pdf.

10. Bassili A, Seita A, Baghdadi S, AlAbsi A, Abdilai I, et al. (2008) Diagnostic and treatment delay in tuberculosis in 7 countries of the Eastern Mediterranean region. Infect Dis Clin Pract 16: 23–35.

11. Fortún J, Martín-Dávila P, Molina A, Navas E, Hermida JM, et al. (2007) Sputum conversion among patients with pulmonary tuberculosis: are there implications for removal of respiratory isolation? J Antimicrob Chemother 59: 794–8.

12. Department of Immigration and Citizenship, Submissions received by the Committee. Inquiry into matters relating to the Torres Strait region. http://www.aph.gov.au/senate/committee/fadt_ctte/torresstrait/submissions.htm, (accessed 5th September 2010).

13. The National Research Institute of Papua New Guinea (2010). Papua New Guinea District and Provincial Profiles. http://www.nri.org.pg/research_divisions/cross_divisional_projects/index.htm (retrieved 30 August 2011).

14. Torres Strait Regional Authority (2010). Torres Strait Regional Authority Annual Report 2009–2010. http://www.tsra.gov.au/media/annual-report-2009-2010/3_Where_We_Operate.pdf (accessed 10 March 2011).
